# Structural determinants of criticality in biological networks

**DOI:** 10.3389/fphys.2015.00127

**Published:** 2015-05-08

**Authors:** Sergi Valverde, Sebastian Ohse, Malgorzata Turalska, Bruce J. West, Jordi Garcia-Ojalvo

**Affiliations:** ^1^ICREA-Complex Systems Lab, Universitat Pompeu FabraBarcelona, Spain; ^2^Institute of Evolutionary Biology (CSIC-UPF), Universitat Pompeu FabraBarcelona, Spain; ^3^Institute of Molecular Medicine and Cell Research, Albert-Ludwigs-Universität FreiburgFreiburg, Germany; ^4^Department of Physics, Duke UniversityDurham, NC, USA; ^5^Mathematical and Information Sciences Directorate, U.S. Army Research Office, Research Triangle ParkNC, USA; ^6^Department of Experimental and Health Sciences, Universitat Pompeu FabraBarcelona, Spain

**Keywords:** criticality, power laws, hierarchical modular networks, neural networks, gene regulatory networks, evolution, robustness

## Abstract

Many adaptive evolutionary systems display spatial and temporal features, such as long-range correlations, typically associated with the critical point of a phase transition in statistical physics. Empirical and theoretical studies suggest that operating near criticality enhances the functionality of biological networks, such as brain and gene networks, in terms for instance of information processing, robustness, and evolvability. While previous studies have explained criticality with specific system features, we still lack a general theory of critical behavior in biological systems. Here we look at this problem from the complex systems perspective, since in principle all critical biological circuits have in common the fact that their internal organization can be described as a complex network. An important question is how self-similar structure influences self-similar dynamics. Modularity and heterogeneity, for instance, affect the location of critical points and can be used to tune the system toward criticality. We review and discuss recent studies on the criticality of neuronal and genetic networks, and discuss the implications of network theory when assessing the evolutionary features of criticality.

## 1. Introduction

The behavior of many complex systems can be described by self-similar mathematical laws. Many of the properties of these systems do not peak around an average value, but display a wide range of values instead. Features as diverse as the distribution of city sizes, the frequency of earthquakes (Gutenberg and Richter, 1944) and of words in English texts (Zipf, 1949), the number of citations received by scientific papers (Yu and Van de Sompel, 1965), and the distribution of income (Mandelbrot, 1963) follow a *power-law*, also known as Zipfs law or Pareto distribution (Schroeder, 1991). In all these systems, we find a power-law defined as *F*(*x*) = *Ax*^−α^, where *A* is a constant and α is the *power-law exponent*, which is an important classification parameter. Any system displaying the power-law behavior has the *scale-free* property, i.e, there is an invariance with respect to scale: any change of scale *C* in the argument of the power-law leaves the shape of the function unaltered. Mathematically, *F*(*Cx*) = *A*(*Cx*)^−α^ = *AC*^−α^*x*^−α^ = *C*^−α^*F*(*x*).

The ubiquity of power laws has created much interest in recent years for several reasons. First, scale invariance can be easily generated with simple generic mechanisms (Sornette, 2006). More importantly, the broad application of power laws is an indication of a deep connection between many unrelated systems. Specifically, all systems having the same power-law exponent share common features in their dynamical processes, and thus their scale invariance must be largely independent of microscopic details (what is known as *universality*). This suggests the remarkable possibility that a general theory can explain the abundance of power laws in a wide array of systems. In statistical mechanics, scale invariance is a well-known, generic feature of *phase transitions* (Yeomans, 1992). The behavior of complex systems can be organized in different types or *phases* separated one from the other by a sharp boundary or phase transition. When the system transitions from one phase to another, it experiences dramatic changes in behavior. At certain (second-order) phase transitions, fluctuations occur at all length scales and the system exhibits scale invariance and power-law behavior.

An important open question is to understand how the system can be tuned to reach (and maintain) this critical state. An elegant (and controversial) theoretical explanation was proposed by Bak et al. (1987) in the framework of self-organized criticality (SOC). The nature of phase transitions results from the interactions between many system components and not from the specific nature of the units (they can be neurons, proteins, species or humans). This motivated Bak and coauthors to investigate simple computational models reproducing the scale-invariance found in real systems. SOC suggests that complex systems can spontaneously evolve toward criticality under a wide variety of situations. The metaphorical visualization of SOC is the flow of sand in a sandpile. The sandpile self-organizes in a minimal stable state because dropping a single grain of sand may set of a large avalanche of activity, which in turn is capable of restoring the previous system stability.

**Figure 1:**
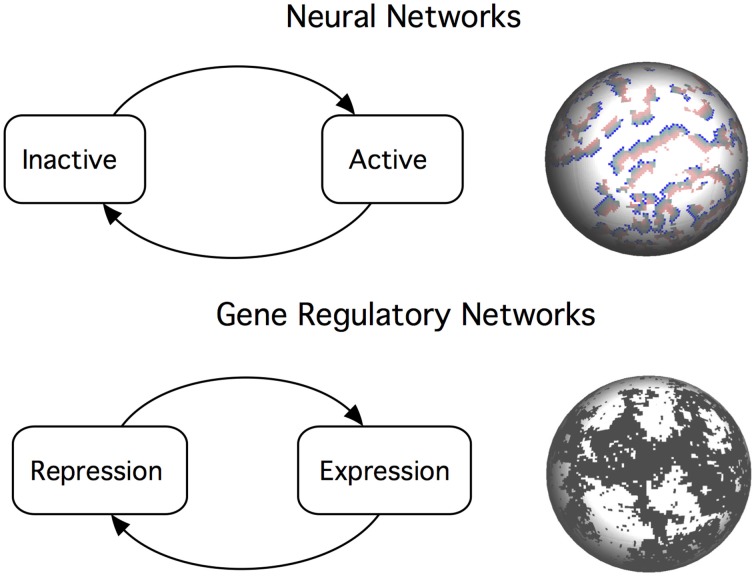
**Critical dynamics in neural networks and gene regulatory networks emerges from a balance between positive and negative interactions between the network components. Top**: An efficient neural network poises itself near the critical point that balances inactivity (death) and chaotic dynamics (epilepsy). This is analogous to the sandpile metaphor: a falling grain dissipates some of its energy to the neighbors, triggering an avalanche of events. **Bottom:** Criticality in gene regulatory networks is described as a boundary between ordered and chaotic dynamics. Switching mechanisms creates complex patterns of gene activation and repression. At the critical point, we observe long-range correlations at all scales.

The sandpile model of self-organized criticality has been criticized for being too simple and unrealistic for biological systems (Gisiger, 2001), and even the behavior of real sandpile has been shown not to be universal (Frette et al., 1996). It is difficult to assess the empirical relevance of SOC because the theory focuses in the dynamics and discards structural features. On the other hand, having a detailed map of any system without any underlying theory of its operation is clearly insufficient. We propose that, in order to fully understand the role played by criticality in biology, we have to extend our theories and fully address the relation between structure and function. Unfortunately, we have only started to study the structure–dynamics relationship in biological systems and there are many open questions. Network studies indicate that the internal organization of biological systems enables the co-existence of different dynamical regimes in the same system. Topological diversity might be an important component when explaining why criticality is observed for a given range of spatial and temporal scales. Here we review recent work on the relationship between structure and critical dynamics in two types of biological networks: neuronal systems and gene regulation (Figure 1).

## 2. Criticality in brain networks

The human brain is considered by many to be the ultimate complex network, in which connectivity spans a wide range of spatial and temporal scales; from the micro level, where an estimated 10^11^ neurons form on average 7000 synaptic connections between neurons, through the meso level of cortical units, and finally to the macro level of specialized brain areas. It is in the last domain where the efficient hierarchical integration of neuronal activity gives rise to the vast repertoire of brain functions, enabling sensory information processing and cognition (Park and Friston, 2013). Integration of discrete short-range synaptic transmissions allows for specialization of functions performed by different neuronal ensembles, while continuous activity of cortical areas demonstrates global synchronization, increasing the effectiveness of interactions between brain regions. Ultimately, the transient nature of configurations of active brain areas reflects the dynamics of cognitive tasks.

Since the neuronal architecture evolves on time scales much longer than the variability of cognitive states, the brain can be viewed as a degenerate system, where multiple functions can be mapped into a fixed anatomical structure. Understanding the emergence of distinctive dynamic states from static connectivity patterns requires that we first understand the relation between the structure and function of brain networks. The statistical physics concept of criticality offers attractive insights into the role structure plays in shaping brain function. In addition it allows us to explain numerous experimental results that capture complex properties of brain dynamics.

The notion of criticality is appealing for many reasons. First, it offers a connection between spatial scales observed in the brain, relating the dynamics at the single neuron level to that registered macroscopically, with fMRI or EEG measurements. As mentioned in the introduction, for a system to be labeled critical its dynamics must be scale-free. As a consequence, it is expected that copies of the system differing in size would behave identically once their size is used as a normalization factor. Consequently, one observes systems at criticality to be dynamically posed between regular or organized dynamics and random behavior, so that they can be maximally responsive to external inputs. To realize such a state, the system is controlled by a tuning parameter, which determines the nature of the dynamics. The cross-correlation between basic units of the system undergoes a transition from being local and short-ranged to spanning the entire size of the system, in analogy to the power-law correlations associated with the divergence of the susceptibility in classical phase transitions. Such long-range scale-free correlations are implicated in superior information transmission properties of systems at criticality (Marinazzo et al., 2014), although some questions have been raised recently on the different behavior of global and local information transfer, with global (collective) information flow peaking outside the critical region in an Ising model (Barnett et al., 2013).

### 2.1. Measures of criticality

In neural systems the most commonly observed signature of criticality is the scaling of the dynamics. First observed by Beggs and Plenz (2003) *in vitro* in slices of rat cortex, the size distribution of neuronal avalanches (corresponding to the number of electrodes in a microelectrode array that are activated simultaneously), was seen to follow an inverse power law with exponent close to 3/2. The avalanche lifetime was also observed to follow an inverse power law with a power-law exponent close to 2. Inverse power-law distributions of neuronal burst sizes have also been observed from sequences of spikes in the isolated leech ganglion (Mazzoni et al., 2007), in dissociated cortical cultures from rat hippocampus (Alessio et al., 2006), in the cortex of awake adult rhesus monkeys (Petermann et al., 2009), and in adult cats (Hahn et al., 2010). This variety of observations suggests that the phenomenon may be quite general. Criticality in brain activity has also been investigated at a larger scale. In particular, in the fMRI recordings of spontaneous activity in healthy subjects, the activity correlation length scales with the functional area size (Fraiman and Chialvo, 2012). Spontaneous brain activity has been also measured in healthy subjects by magnetoencephalography (MEG), and found to scale with the same exponent as neuronal avalanches (Shriki et al., 2013).

Despite mounting experimental results indicating that neural systems are posed near a critical state, the straightforward adoption of the physical notion of criticality in the context of a biological system might be premature, or it might even be wrong. One needs to remember that the presence of scaling in a process is not sufficient to declare it critical. Only a limited number of studies (Beggs and Plenz, 2003) have investigated other aspects of criticality, such as how the dynamics changes as system size varies, while others report lack of system-wide activity (Shriki et al., 2013), or the absence of scaling behavior all together (Dehghani et al., 2012). The critical point in physical sciences originates from the fact that a system's dynamics is preserved across multiple scales. In the brain however, the nature of synaptic activity is fundamentally different from the quasi-periodic activity of brain regions. The integration of local activity along the hierarchical structure of the brain incorporates active processing of information, not simply the linear summation of signals (Barardi et al., 2014). Additionally, the measured macroscopic properties of the brain often arise from analysis of functional activity, while microscale recordings sample the dynamics of actual neuronal architecture. Finally, the physical notion of a control parameter that requires tuning for criticality to emerge, finds no clear correspondence in the control of brain dynamics. Existing approaches lack precise physiological descriptions for how the brain maintains its tuning near a critical point. In a recent paper, Roberts et al. (2014) argue that a key ingredient missing is a formulation of reciprocal coupling between neural activity and metabolic resources. At the same time the balance between excitation and inhibition (Hobbs et al., 2010; Malagarriga et al., 2015) or variability in connection strengths (Chen et al., 2010) have been suggested as possible mechanisms controlling the dynamical state of neural network.

### 2.2. Structural determinants

In the absence of undisputed experimental evidence of the brain being a critical system, the neuroscience community has adopted computational methods in an attempt at identifying mechanisms responsible for critical behavior. One question in that direction is the role that neuronal network structure plays in its dynamics. Since the detailed organization of the human brain network across all scales is not yet experimentally accessible, most studies concentrate on the properties of large-scale networks. Diffusion magnetic resonance imaging (dMRI) allows us to asses the structural connectivity by identifying tracks of axon bundles as they traverse the brain's white matter. By contrast, functional MRI (fMRI) allows us to estimate functional connectivity between brain regions by measuring variability of the blood-oxygenation-level-dependent (BOLD) signal in the gray matter regions, as they participate in performance of specific cognitive tasks. Since both techniques are strikingly different in what they attempt to measure and in how they measure it, the resulting connectivity patterns cannot be treated as equivalent. In non-human primates the comparison between functional and anatomical connections has revealed striking similarities (Margulies et al., 2009). In humans, however, while the strength of structural connectivity partially predicts the strength of functional connections, the knowledge of functional network generally does not allow the investigator to determine the underlying structural topology (Honey et al., 2009).

Modern network science has revealed fundamental aspects of normal brain network organization, such as small-world and scale-free patterns, hierarchical modularity, hubs and rich clubs.

#### 2.2.1. Small-world behavior

The small-world topology has been found systematically in structural (Hagmann et al., 2007; He et al., 2007) and functional brain networks (Eguiluz et al., 2005), and it has also been identified at the cellular scale in cortical neuronal circuits in mammals (Yu et al., 2008) and even in the nervous system of the perfectly mapped organism *C. elegans* (Watts and Strogatz, 1998). Characterized by a combination of high clustering and short path length, the small-world architecture offers an attractive view of the brain, since it provides a balance between the segregation and integration of information and offers a solution to the conflicting constraints of reducing wiring costs and facilitating information flow.

#### 2.2.2. Scale-free topology

Brain networks demonstrate broad degree distributions (Eguiluz et al., 2005), implying vast differences in connectedness and centrality between the nodes. In particular the presence of highly connected subsets of nodes (rich clubs) has been detected (van den Heuvel and Sporns, 2011) and discussed as offering resilience to random node removal. Healthy brains display also a hierarchical modular structure (Meunier et al., 2010) characterized by self-similarity, giving rise to fractal modularity (Gallos et al., 2012). Traditionally, the link between topological structure of brain networks and their dynamical properties is assessed by explicitly assuming a specific structural network architecture (e.g., random, small-world, or scale-free). The dynamics of an integrate-and-fire (IF) neural model simulated on a fully connected neural network demonstrated an inverse power-law distribution of avalanche sizes (Levina et al., 2007), characterized by the same exponent as *in vitro* observations. Similar results were recovered for a simplified IF model with constant synaptic strengths (Choi et al., 2014) evaluated on random and small-world networks. The comparison of scale-free, random and small-world architectures with the Izhikevich model of neuronal dynamics (Massobrio et al., 2013) show that scale-free and small-world topologies display scaling behavior characteristic of criticality.

#### 2.2.3. Hierarchical modularity

With the discovery of the hierarchical nature of brain networks (Gallos et al., 2012), the role of modules became a central focus. Hierarchical modular networks (HMN) are obtained by modification of a random topology, where the initial network is first divided into a specified number of modules, and connections between modules are rewired with preference for a specific module. Simulations of a random neural network model (Vogels and Abbott, 2005) on an HMN topology demonstrated sustained activity characterized by inverse power-law distributions of burst activity (Wang et al., 2011). In contrast with a single densely connected module, which is unable to sustain spontaneous activity, connections between modules served as a source of external noise for each of subsystems, giving rise to sustained neuronal firing. However, as in the case of simple topologies, the results seem to be model dependent, since a modular network implementing statistical features observed in human brain (Russo et al., 2014) did not lead to fully scale-free activity (Levina et al., 2007). In fact, the avalanches invaded different modules but were able to activate only a few neurons in each one. Critical behavior spanning the whole network was recovered when the number of inter-modular connections was increased, modifying the modular structure into random network.

**Figure 2:**
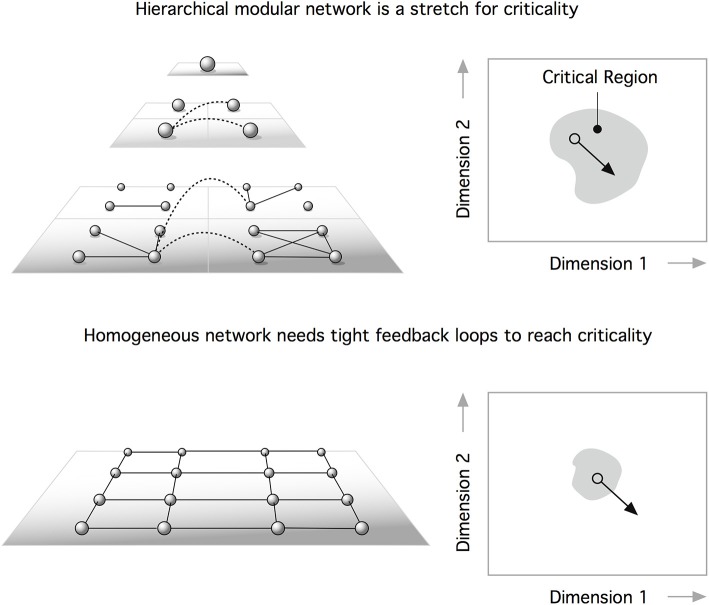
**The existence of feedback loops can self-organize neural networks at the critical point**. One problem is that perturbations can displace the system outside the critical domain **(bottom)**. According to Moretti and Muñoz (2013), a hierarchical modular network is a path to stretched criticality. This means that a heterogeneous architecture can extend criticality from a critical point to a critical region. This extended parameter range for critical behavior makes hierarchical systems **(top)** more robust to perturbations than homogeneous networks (bottom).

Recently, Moretti and Muñoz (2013) suggested a dramatically different interpretation of the role that the hierarchical structure of brain networks plays in their dynamics. Perplexed by the rich set of functional signatures identified in functional brain networks (Damoiseaux et al., 2006), which demonstrate temporal variations (Schaefer et al., 2014; Zalesky et al., 2014), Moretti and Muñoz argued for an interpretation of brain dynamics as a system operating not at a precise, fine-tuned critical point, but rather a whole extended region around it. Adopting the framework of HMN (Moretti and Muñoz, 2013), the authors demonstrated that modularity leads to behavior where subcritical and supercritical dynamics coexist, being present in different modules at the same time (Figure 2). The presence of active regions, even if the system is globally in a disordered state, leads to an anomalous behavior, reminiscent of Griffiths phases (Griffiths, 1969).

## 3. Criticality in gene networks

Mammalian neuronal networks contain on the order of 10^9^ nodes, whereas gene networks operate on a much smaller scale, of the order of 10^4^ nodes (Hecker et al., 2009). In this section we describe the peculiarities of gene networks, introduce the framework of random Boolean networks and review empirical and analytical measures of criticality that have been applied to this model system. Subsequently, we highlight known relations between critical dynamics and network structure and explore their evolutionary origins.

Krotov et al. (2014) describe four dynamical signatures of criticality derived from a simple two-gene system: (i) strong correlations between the fluctuations of the activity of different nodes, (ii) temporal correlations in node activity trajectories (associated with a slowing down of the dynamics), (iii) spatial correlations of node fluctuations across long distances, and (iv) deviation of these fluctuations from a Gaussian distribution. Unfortunately direct observation of these signatures at sufficient temporal resolution and accuracy is currently unfeasible. The structural characterization of gene networks is also still far from complete, due to a number of challenges that include the sheer complexity of the system and in particular the curse of dimensionality (Hecker et al., 2009), which have hampered reverse-engineering efforts to date. Similarly to the case of brain networks discussed above, past research at the intersection of gene networks and criticality has therefore focused on simulations, guided by prior biological knowledge and by the empirical evidence at hand. In part, such evidence consists of high-throughput gene expression measurements and gene interaction networks compiled from previous studies (Serra et al., 2004; Shmulevich et al., 2005; Nykter et al., 2008; Balleza et al., 2008; Pomerance et al., 2009; Krotov et al., 2014). In the literature, generative models of gene network structure and node dynamics differ in complexity, biological realism, and in the way criticality is measured. However, only the most idealized simulations give rise to ordered or chaotic dynamics. Most authors suggest that gene networks operate close to criticality (Kauffman, 1969; Fox and Hill, 2001; Aldana, 2003; Rämö et al., 2006; Nykter et al., 2008; Torres-Sosa et al., 2012; Krotov et al., 2014). However, it is difficult to exclude the possibility that this outcome is due to publication bias, and in any case how “close” to critical dynamics the different systems are is not well defined.

### 3.1. Random boolean networks

A vast majority of studies on gene network dynamics have been conducted within the framework of random Boolean networks (RBNs). This framework was introduced in the late 1960s by Stuart Kauffman, with the specific aim to study the properties of gene networks (Kauffman, 1969) (see the article by Drossel, 2008 for a comprehensive review). Briefly, random Boolean networks are a type of complex network with a limited set of allowed node states and transfer functions^1^. The state of each node (gene) is restricted to only two possibilities, on or off. Formally, a Boolean network is a directed graph Ω(*V, E, B*) with a set of Boolean functions *B* = {*b*_*i*_|*i* = 1 … *n*} such that *b*_*i*_ : {0, 1}^*k*^ → {0, 1}, with *k* ≤ *n*. Before a simulation in this framework is initiated, a random initial state is set for each node. During the simulation, the state of a node at time *t* is given by *x*_*i*_(*t*), and the next state after each iteration is given by *x*_*i*_(*t* + 1) = *b*_*i*_(*x*_*i*1_(*t*), *x*_*i*2_(*t*) … *x*_*ik*_(*t*)), where *x*_*ij*_ are the states of the nodes connected to node *i*. The states of all nodes are updated simultaneously according to this rule. This process may be iterated until convergence to a stable fixed point or limit-cycle. The simplification provided by a random Boolean network model enables a systematic exploration of the relationship between network structure and critical dynamics that might otherwise be unfeasible.

### 3.2. Measures of criticality

In its most simple realization, each node of a Boolean network is connected at random to a set of *K* input nodes, and one chooses uniformly at random a possible transfer function. In such a homogeneous network the median cycle length is 0.5 · 2^*N*/2^ (Legenstein and Maass, 2007). Due to the finite size of the network its convergence is guaranteed. The Hamming distance measures the minimum number of substitutions to convert one Boolean network state into another, and is used to measure the evolution of the system at each iteration. Formally:

$$H(t)=\sum_{i\;=\;1}^{n}|x_{i}(t)-{\tilde{x}}_{i}(t)|,$$

where *x* and $\tilde{x}$ are two slightly different initial states of the same network, and *i* runs over all nodes of the network (Pomerance et al., 2009). As the number of iterations tends to infinity in a finite network, *H*(*t*) → 0, but does so more slowly the more erratic the behavior is. In the limit of infinite size the network can become chaotic. The slope of the *H*(*t*) curve at the origin is indicative of criticality. This is an empirical measure (Legenstein and Maass, 2007). According to this measure and under the annealed approximation (through which all Boolean functions are randomized at each iteration), the dynamics becomes critical for *K* = 2, whereas networks with *K* = 1 operate in an ordered regime.

The analytical definitions of criticality are increasingly generalized to allow application to more realistic and complex models of gene regulatory networks. Shmulevich et al. (2005) generalized the initial formula to allow computation for the case where network functions are generated according to probability distributions that favor some variables over others, measured through their activities, or when transfer functions are chosen at random from certain classes (such as canalizing functions). Pomerance et al. (2009) generalized the initial formula to allow (i) any network topology, (ii) a distribution of biases instead of one parameter, (iii) non-synchronous updates, and (iv) multiple node states, while still permitting the calculation of the control parameter at which the network dynamics is critical. The method uses the maximum eigenvalue of a modified adjacency matrix. In any case, it must be noted that the concept of criticality loses its utility without a clear definition of how close to the critical threshold network dynamics must be in order to qualify as critical.

### 3.3. Structural determinants

A major aim in the literature has been to demonstrate the phenomenon of criticality in specific gene networks, which have been inferred from (incomplete) empirical evidence (Shmulevich et al., 2005; Balleza et al., 2008; Nykter et al., 2008). The question of how structural features contribute to the emergence of criticality remains largely unaddressed. Here we give an overview on what is known of the effect of structural properties on the location of critical points within the framework of the random Boolean network model, discussing in particular the case of scale-free architectures and the roles of community structure and canalizing functions.

#### 3.3.1. Scale-free topology

Aldana et al. (2007) argue that a scale-free topology diminishes the need to fine-tune connectivity parameters (the rewiring probability *p* and the average degree *K*) to obtain critical dynamics. In particular, the critical phase transition in scale-free networks occurs over a range of scale-free exponents (α ∈ [2.0, 2.5]) and allows for a range of connectivities. Along this line, Fox and Hill (2001) argue that homogeneous topologies with biologically realistic connectivities would lie in the chaotic regime, since their average connectivity (measured by *K*) is relatively high. If gene networks indeed operate at criticality, a scale-free topology might explain this discrepancy. In the thermodynamical limit, broad degree distributions do not affect the critical point (provided *K* is fixed), but in finite settings power-law distributions lead to increased order. For example, even if the average *K* is large in a given network, there can be many nodes with low in-degrees that are likely to be frozen nodes. This reduces the size of the network that is active and effectively involved in the dynamics, which in turn reduces the real value of average *K* for the network, since many of those in-degree links might come from frozen connections, and thus do not contribute to potentially chaotic dynamics (Fox and Hill, 2001).

#### 3.3.2. Modularity

The presence of community structure in the network difficults signal transmission, pushing the system into an ordered phase (Wang and Albert, 2013). Also, modularity broadens the range of connectivities which allows for critical dynamics. Modular RBNs have more attractors and are closer to criticality when chaotic dynamics would be expected, compared to classical RBNs (Poblanno-Balp and Gershenson, 2011). In general, modules make it difficult for damage to spread through the network, even if the local connectivity (within a module) is high. In this way, chaotic dynamics can be constrained within modules (Gershenson, 2012). In contrast with the effects described above, modularity also allows for information flow between modules, and thus while reducing the occurrence of chaos it might also contribute to the spreading of the critical regime, much like Griffiths phases (Hesse and Gross, 2014) (see above), because modules are often connected with each other, leading to a small-world topology which in turn allows for more critical dynamics. Lizier et al. (2011) argue that a small-world topology in RBNs has relatively large information storage and transfer capabilities, and enables critical dynamics.

#### 3.3.3. Transfer functions

The effect of varying the rewiring probability *p* or their in-degree *K* can be replicated by changing the incidence of canalizing functions (Shmulevich and Kauffman, 2004), which yield dominating inputs in transfer functions (so that the node would be unaffected by other inputs). Canalizing functions are found with high probability when selecting Boolean functions uniformly at random (Serra et al., 2004), and are thought to occur in realistic gene regulatory networks (Shmulevich and Kauffman, 2004). Balleza et al. (2008) used networks from several model organism networks to argue that increasing the probability of canalizing functions, while generally pushes dynamics toward the ordered phase, is not sufficient to leave the critical regime. The results are essentially the same if the fraction of canalizing functions is not inferred from the microarray data (Balleza et al., 2008). The effect may be similar to silencing, the fixation of a subset of nodes in a particular state, which has been shown to make the system more ordered (Luque and Solé, 1997; Serra et al., 2004).

### 3.4. Evolutionary mechanisms

**Figure 3:**
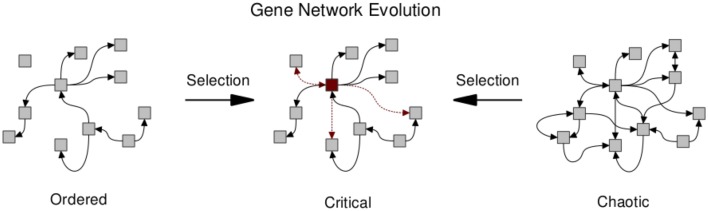
**Natural selection pushes gene regulatory networks toward the critical regime due to the opposing forces of conserving essential network function and allowing for the evolution of potentially beneficial modifications**. Arrows between nodes denote regulatory interactions. Those retained or gained under selection are highlighted (red dashed). The manifestation of hub-like nodes (red square) has been observed under simulations of network evolution (Torres-Sosa et al., 2012).

Several works have investigated the evolutionary mechanisms leading to network structures that may in turn facilitate critical dynamics (Bornholdt and Rohlf, 2000; Solé and Valverde, 2006, 2008; Aldana et al., 2007; Torres-Sosa et al., 2012). Criticality in gene regulatory networks may in fact be ubiquitous due to evolutionary mechanisms (see Figure 3). Biological networks are subject to an evolutionary trade-off between conserving essential network function while allowing for modifications that may increase fitness. Clearly, any system replicating and competing under natural selection must be able to conserve current functions; but also needs to be able to adapt. Given these two constraints, Torres-Sosa et al. (2012) simulate the evolution of gene regulatory networks in the random Boolean framework described above, under a fitness function that penalizes the loss of existing attractors and rewards the creation of novel attractors. Specifically, gene regulatory interactions are mutated and grown by the mechanism of gene duplication. Network instances are selected to maintain their current dynamical attractors (i.e. their current phenotypes) while generating new ones. The authors show that the selected networks display criticality (Figure 3). However, it should be noted that to produce non-trivial networks it is necessary to introduce an α-fitness criterion, which prescribes a low fitness to nodes that are always frozen and thus have a minimal dynamic range.

Another example showing how standard evolutionary mechanisms lead to critical dynamics was given by Bornholdt and Rohlf (2000). The selection rules used in that case were such that nodes that do not change their state within the attractor trajectory receive new connections at every iteration. This leads to an average connectivity of the network equal to the critical connectivity, without the need of tuning the system. In this way this process leads to self-organization of the network in terms of its average connectivity. A similar conclusion was reached by Aldana et al. (2007).

## 4. Outlook

The concept of critical dynamics has been a fundamental principle in statistical physics for decades. Recent years have witnessed substantial efforts to extend this framework to biological networks. However, multiple differences exist between biological networks and classically studied physical systems. These differences include the heterogeneity of the structure and composition of biological networks, the lack of equilibrium or near-equilibrium states, the existence of multiple feedback loops that serve as potential tuning parameters, and the presence of multiple time scales. All these differences suggest that the notion of criticality as defined by statistical physics could be insufficient in the context of truly complex systems such as the brain. Accordingly, authors such as Longo et al. (Bailly and Longo, 2011; Longo and Montévil, 2012) have argued for a novel form of criticality, intrinsic to biological systems. Features typically associated with criticality, such as long-range correlations, are being observed in biological systems including both brain and gene networks. The potential of long-range correlations for leading to traits that are inherently beneficial to biological systems, such as information processing and evolvability, is undeniable. We need to reach a complete understanding of how such dynamical traits emerge from the specific structural properties (such as modularity and topological heterogeneities) of biological networks.

### Conflict of interest statement

The authors declare that the research was conducted in the absence of any commercial or financial relationships that could be construed as a potential conflict of interest.

## Footnotes

^1^Transfer functions integrate the signal from all incoming edges to a particular node to determine the node state at time *t*.
